# Correlation between apelin and VEGF levels in retinopathy of prematurity: a matched case–control study

**DOI:** 10.1186/s12886-022-02565-x

**Published:** 2022-08-11

**Authors:** Yimin Zhang, Jing Feng, Shuming Shao, Qing Mu, Jie Liu, Chaomei Zeng, Xiaorui Zhang

**Affiliations:** 1 Department of Pediatrics, Peking University People’s Hospital, No. 11 Xizhimen South Street, Xicheng District, Beijing, 100044 People’s Republic of China; 2 Department of Ophthalmology, Beijing Chaoyang Hospital, Capital Medical University, Beijing, China; 3 Department of Central Laboratory & Institute of Clinical Molecular Biology, Peking University People’s Hospital, Beijing, China

**Keywords:** Apelin-13, Vascular endothelial growth factor, Retinopathy of prematurity, Preterm infant

## Abstract

**Background:**

Although several clinical studies have analysed the relationship between the levels of vascular endothelial growth factor (VEGF) and apelin-13 in venous blood and retinopathy of prematurity (ROP), no definitive conclusions have been reached. This study aimed to investigate the relationship between apelin-13 levels and VEGF levels and ROP.

**Methods:**

Differences in plasma apelin-13 and VEGF levels were analysed in two groups of infants born with birth weight < 1500 g and gestational age < 32 weeks at Peking University People’ s Hospital. One group comprised infants diagnosed with ROP and the other group was a control group comprising infants without ROP.

**Results:**

Apelin-13 levels were significantly lower in the ROP group than in the control group, while VEGF levels showed the opposite result (both *P* < 0.001). Infants with severe ROP had lower apelin-13 levels and higher VEGF levels than with mild ROP (both *P* < 0.05).The receiver operating characteristic curve for apelin-13 level as the indicator of ROP showed that a cut-off value of 119.6 pg/mL yielded a sensitivity of 84.8% and a specificity of 63.6%, while for VEGF level, the cut-off value of 84.3 pg/mL exhibited a sensitivity of 84.8% and a specificity of 66.7%.

**Conclusions:**

Plasma apelin-13 and VEGF levels at 4–6 weeks of age may play a role in assisting the diagnosis of ROP.

## Background

Retinopathy of prematurity (ROP) was first reported in 1942, and since then, it has attracted widespread attention. With the wide application of ventilators and pulmonary surfactants, the survival rate of preterm infants has increased significantly, and consecutively, the incidence of ROP has also increased. ROP is the most important cause of blindness in children worldwide [1], and therefore, early detection and treatment of ROP are very important.

The pathophysiology of ROP can be divided into two stages. The first phase involves cessation of vessel growth and some loss of the already formed vessels, which is driven by the higher than normal physiologic intrauterine blood oxygen levels associated with premature birth. The second phase occurs when the avascular retina becomes more metabolically active; pathologic neovascularisation, which carries a potential for retinal detachment, then follows. Most scholars believe that the second stage is jointly regulated by vascular endothelial growth factor (VEGF), which is produced by the ischaemic retina, and other angiogenic factors, resulting in typical abnormal angiogenesis in ROP [2]. Several clinical studies have analyzed the relationship between ROP and systemic VEGF concentrations; however, the reported findings have been inconsistent. Some studies have shown that the level of VEGF in venous blood is not related to the occurrence of ROP; whereas, others have reported contradictory findings that preterm infants with ROP have lower blood VEGF levels [3] and that circulatory concentrations of VEGF were elevated in infants with ROP [4].

In terms of angiogenic factors, apelin is a recently discovered angiogenic factor, which together with the angiotensin I receptor-related protein, APT, forms the apelin/APJ system. This system is widely distributed and expressed in various organs of the human body, and there are several active formulations of this system consisting of 12, 13, 15, 16, 17, 19, 28, 31, and 36 amino acid residues as well as a pyroglutamate apelin-13 form [5]. All bioactivity is thought to be in the terminal 13 aa fragment (apelin 13) [6]. In recent years, oxygen-induced neonatal retinopathy mouse experiments have suggested that the expression of apelin-13 can affect vascular density and morphology [7, 8]. Furthermore, it may play an important role in pathological retinal neovascularisation and affect the occurrence and development of ROP. However, few in vivo studies have analyzed the relationship between ROP and systemic apelin-13 concentrations [9]. Moreover, the relationship between apelin-13 and VEGF is unclear. In vitro studies have found that apelin-13 can promote neovascularisation by up-regulating the level of VEGF, suggesting that the apelin/APJ pathway may have a synergistic effect with VEGF [10], while other experiments have found that apelin-13 antagonists can prevent pathologic retinal formation without affecting the expression level of VEGF, supporting the hypothesis that apelin-13 and VEGF are independent of each other in pathological angiogenesis [7]. Moreover, there are limited in vivo studies in the current literature. Therefore, the relationship between apelin-13 and ROP and the expression of apelin-13 and VEGF in infants with ROP remains unclear.

Based on the inconsistent previous findings, this study aimed to analyze the association between systemic apelin-13 and VEGF concentrations and ROP and explore the correlation between the abovementioned cytokines to provide a basis for the development of future ROP screening methods.

## Methods

### Study design and population

This was a prospective case-control study conducted in the ophthalmology clinic and paediatric clinic of Peking University People’s Hospital. The study applied the ROP screening guidelines of the UK [11]. All participants had a birth weight < 1500 g and gestational age < 32 weeks and underwent regular ophthalmological screening from January 2016 to January 2018. Participants with a chromosomal or major congenital anomaly, those without blood samples, and those who declined participation or were lost to follow-up were excluded. This study was performed in accordance with the Declaration of Helsinki and was approved by the local ethics review board (2015PHB108-01). Written informed consent was obtained from the parents or legal guardians of included patients.

Ophthalmologic examination for ROP was performed between 4 and 6 weeks of age and repeated after 1–2 weeks according to the examination results until the retinal vascularisation was well into zone III. The presence or absence of ROP and other diseases and zone, stage, and extent of ROP were recorded according to the international ROP classification (stages 1–5). The ROP group comprised infants with any ROP stage. The control group comprised infants without ROP who were randomly matched with the ROP group 1:1 by gestational age and birth weight. Infants with ROP were divided into mild and severe groups. We used the term ‘severe ROP’ to include patients treated for type 1 pre-threshold and threshold disease and those presenting with stages 4 and 5 ROP, according to the international classification system (stage 1: demarcation line; stage 2: ridge; stage 3: extraretinal fibrovascular proliferation; stage 4: partial retinal detachment; and stage 5: total retinal detachment) [12].

At registration in the ophthalmology clinic or paediatric clinic, information on infants' demographic characteristics (e.g. gestational age and birth weight) and post-natal illness (e.g. post-natal Apgar score, history of oxygen inhalation, and history of blood transfusion) were recorded.

### Sample collection and measurement

Samples were collected when blood was drawn for other purposes from a patent line during the period of 4–6 weeks after birth (at the time of the first ophthalmological examination) for measurement of plasma apelin-13 and VEGF levels. Venous blood samples (0.5 mL) were collected in tubes containing ethylenediaminetetraacetic acid and centrifuged for 10 min at 3,000 rpm. Then, the plasma was separated. The blood was stored at -80 °C in polypropylene tubes until the time of the assay. All samples from an individual infant were analysed using the same assay. The blood levels of apelin-13 peptide and VEGF were measured using enzyme-linked immunosorbent assay kits (RayBiotech, Inc., Norcross, GA, USA) [13]. All the samples were diluted two-fold with the diluent provided with the system and were analyzed in duplicate according to the manufacturer’s instructions. The optical density was determined at 450 nm using an absorption spectrophotometer. Each sample was analyzed in duplicate, and the mean value of the two readings was used for quantitative analysis.

### Statistical analyses

Statistical analyses were performed using SPSS Statistics 23 (IBM Corp, Armonk, NY, USA) and GraphPad Prism 8.2.0 (GraphPad, San Diego, CA, USA). Continuous variables, expressed as mean (standard deviation) or median (interquartile range), were compared using Student’s t-test or Mann–Whitney U test. Categorical variables, presented as count (percentage), were compared using the χ^2^ test or Fisher’s exact test for small cell counts (*n* < 5). A *P* value of < 0.05 was considered to indicate a statistically significant difference. Receiver operating characteristic (ROC) curves were plotted to determine the potential of apelin-13 and VEGF expression to differentiate ROP group samples from control group samples. Pearson’s correlation test was used to assess the correlation between apelin-13 and VEGF in infants.

## Results

### Population

**Fig. 1:**
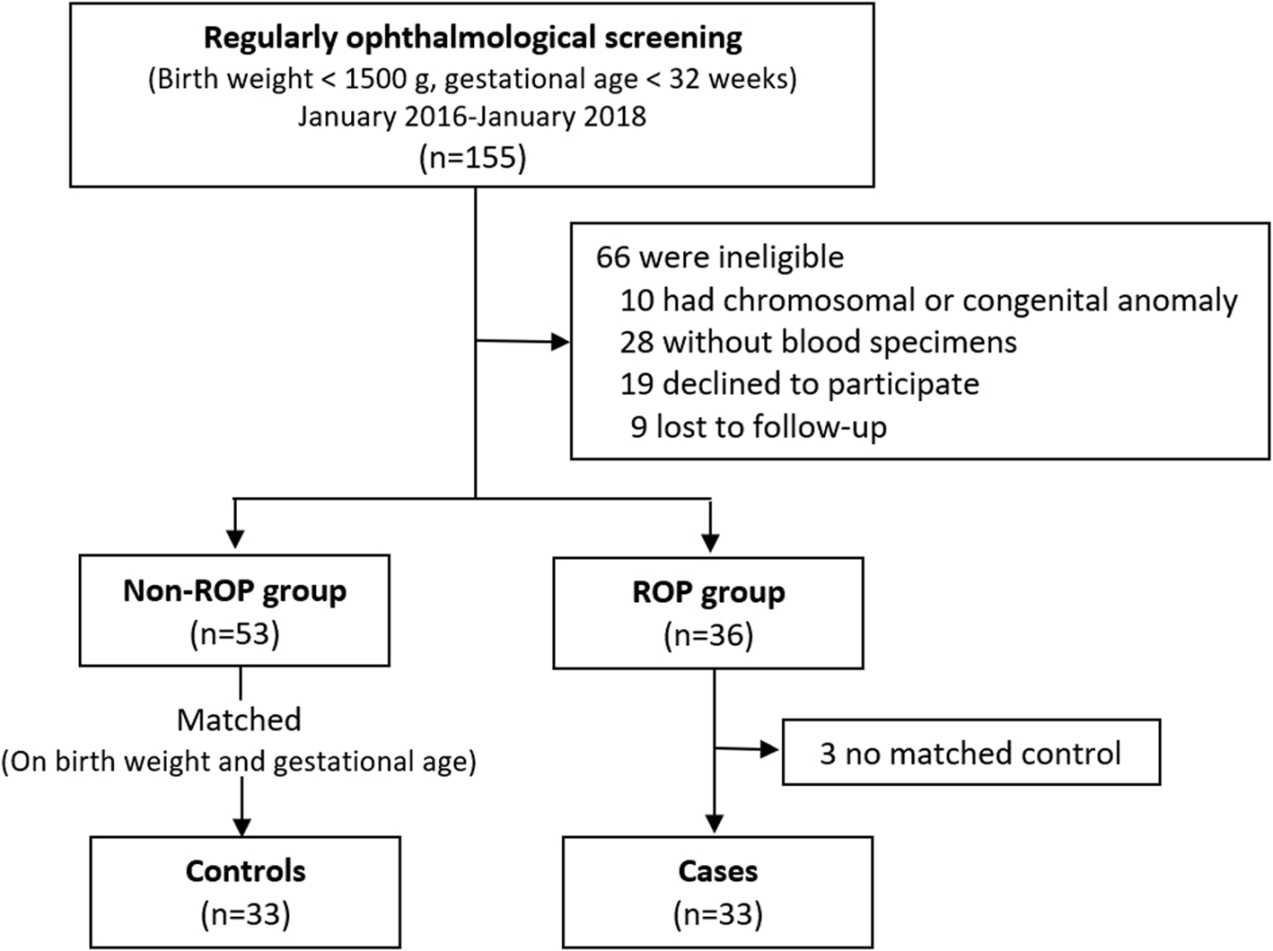
Flow chart of the study

A total of 66 participants, including 33 in the ROP group and 33 in the control group, were included in the final analysis (Fig. 1). The baseline characteristics were similar in both groups of infants (Table 1). There were no significant differences in gestational age, birth weight, Apgar score, oxygen supplementation history (all infants had a fraction of inspiration below 40%), or blood transfusion history between the two groups.

**Table 1:** Perinatal characteristics of babies eligible for the case–control study

Perinatal characteristics	ROP group (*n* = 33)	Control group (*n* = 33)	*P*-value
Gestational age(weeks) (Mean ± SD)	29.5 ± 2.0	29.4 ± 2.0	0.803
Boys	16 (48.5)	16(48.5)	1.000
Birth weight (g) (Mean ± SD)	1206.1 ± 279.8	1128.8 ± 255.6	0.246
Small for gestational age	4 (12.1)	4 (12.1)	1.000
Multiple pregnancy	8 (24.2)	6 (18.2)	0.547
Apgar score (Mean ± SD)			
1 min	7.2 ± 1.3	7.2 ± 1.2	0.921
5 min	8.8 ± 0.8	8.9 ± 0.7	0.507
Perinatal asphyxia	13 (39.4)	15 (45.5)	0.686
oxygen supplementation			
< 7 days	8 (24.2)	12 (36.4)	0.284
> 7 days	22 (66.7)	17 (51.5)	0.211
Blood transfusion	18 (54.5)	17 (51.5)	0.805

*ROP* Retinopathy of prematurity

### Correlation between apelin-13 and VEGF levels

**Fig. 2:**
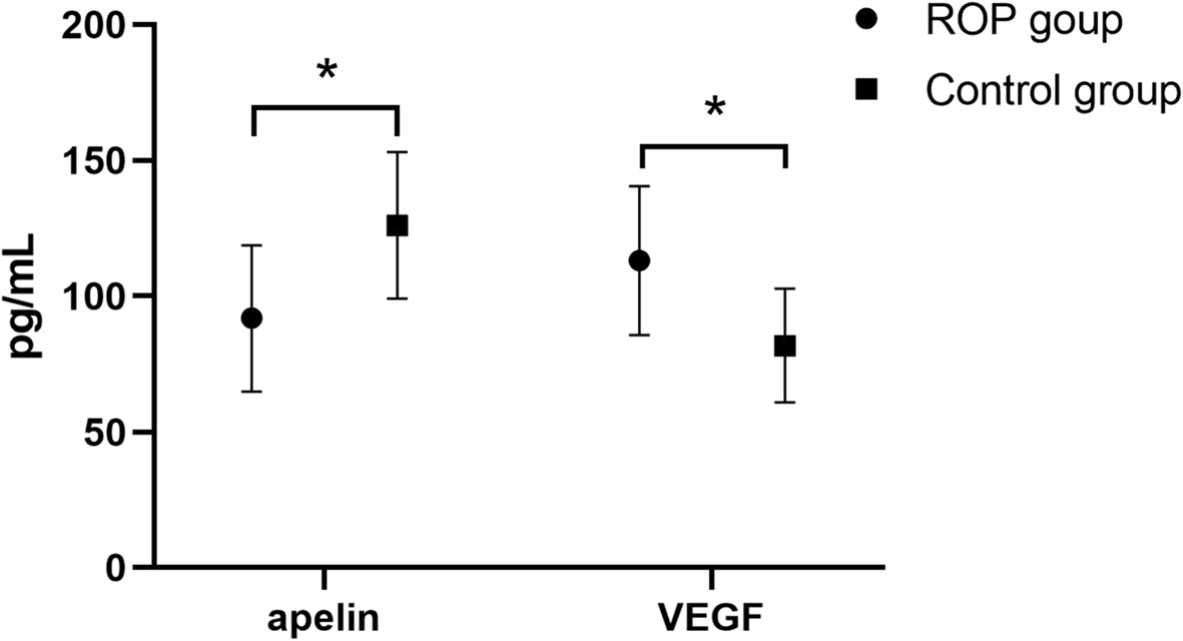
Comparison of blood apelin-13 and VEGF levels in the ROP group and control group. *P < 0.05. ROP, retinopathy of prematurity; VEGF, vascular endothelial growth factor

**Fig. 3:**
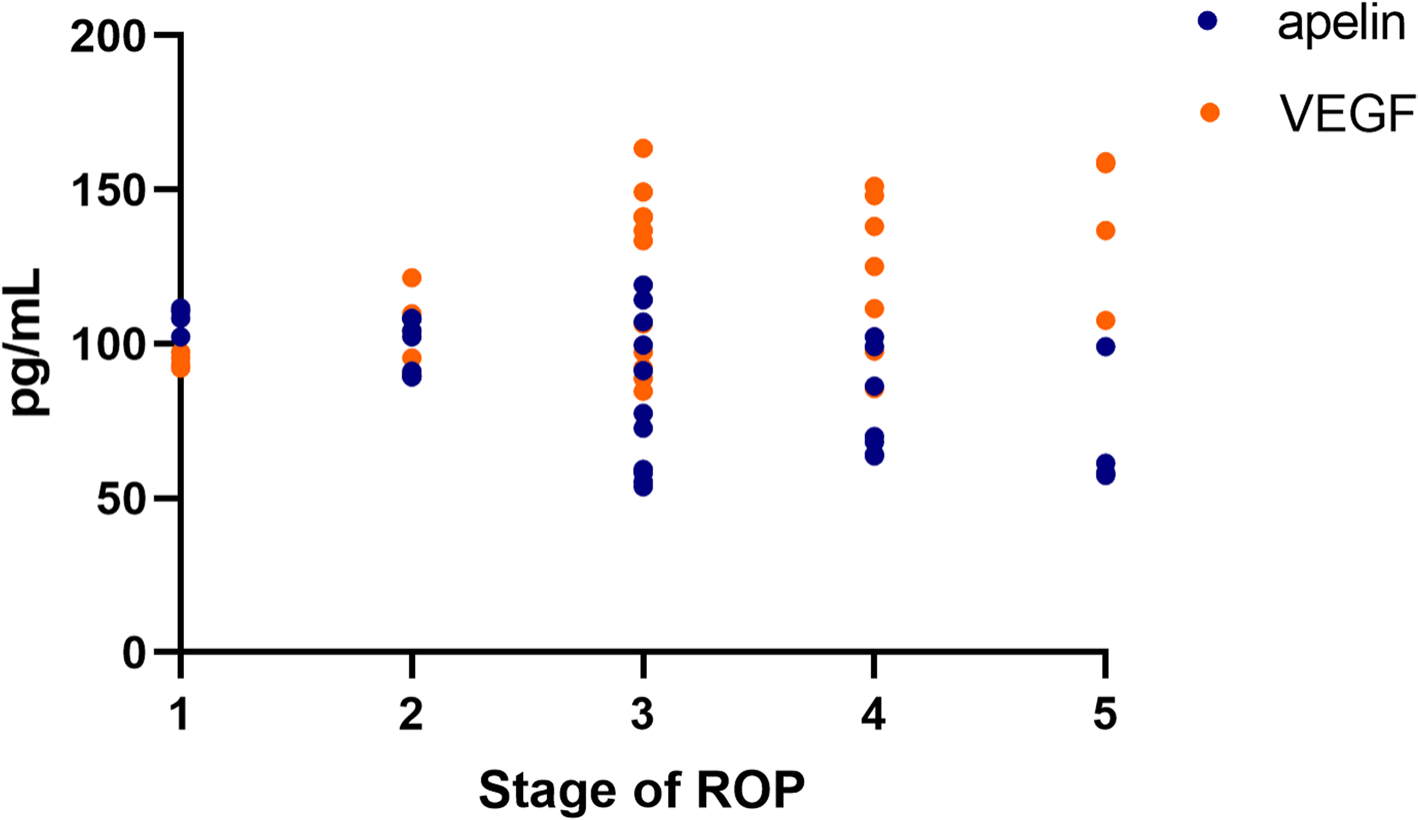
Scatterplot showing the association between apelin-13 and VEGF levels in ROP. ROP, retinopathy of prematurity; VEGF, vascular endothelial growth factor

**Fig. 4:**
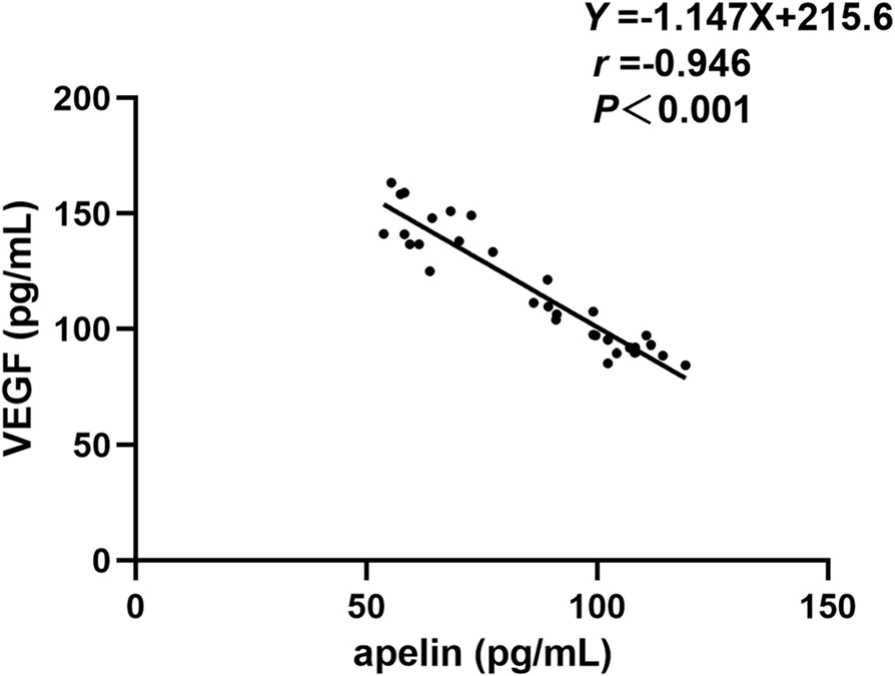
Scatterplot showing the association between plasma VEGF and apelin-13 levels in infants with ROP, with a statistically significant negative correlation between the parameters (*r* = 0.931, *P* < 0.001). ROP, retinopathy of prematurity; VEGF, vascular endothelial growth factor

**Fig. 5:**
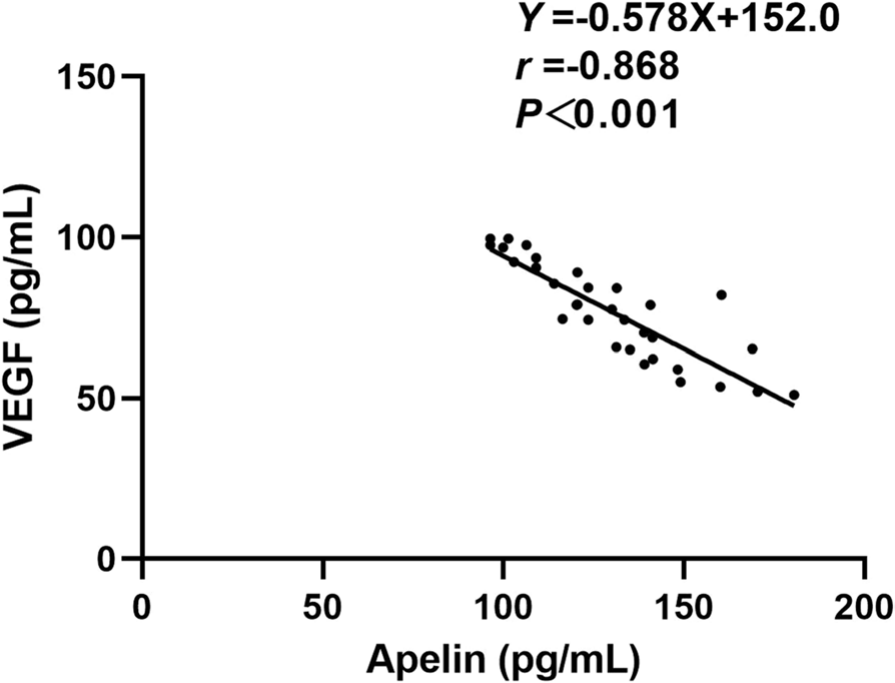
Scatterplot showing the association between plasma VEGF and apelin levels in infants without ROP, with a statistically significant negative correlation between the parameters (*r* = 0.868, *P* < 0.001)

Plasma apelin-13 levels were significantly lower in the ROP group (91.8 ± 26.9 pg/mL) than in the control group (126.0 ± 27.0 pg/mL), while plasma VEGF levels were significantly higher in the ROP group (113.1 ± 27.3 pg/mL) than in the control group (81.8 ± 20.9 pg/mL) (both *P* < 0.001; Table 2; Fig. 2). Moreover, lower plasma apelin-13 levels were correlated with higher stages of ROP (75.4 ± 17.7 pg/mL versus 92.4 ± 20.7 pg/mL) and higher plasma VEGF levels were correlated with higher stages of ROP (129.0 ± 25.4 pg/mL versus 109.7 ± 23.7 pg/mL) (both *P* < 0.001; Table 3). The scatter plots of apelin-13 and VEGF levels at different ROP stages are shown in Fig. 3. Pearson’s linear correlation analysis using apelin-13 level as the dependent variable and VEGF level as the independent variable showed a negative correlation between apelin-13 and VEGF levels in infants with and without ROP (*r* = 0.946, *P* < 0.001 and *r* = 0.868, *P* < 0.001, respectively; Figs. 4 and 5).

**Table 2:** Plasma apelin and VEGF levels at 4–6 weeks among the studied groups

Levels	ROP group (*n* = 33)	Control group (*n* = 33)	*P*-value
apelin (pg/mL)	91.8 ± 26.9	126.0 ± 27.0	< 0.001*
VEGF (pg/mL)	113.1 ± 27.3	81.8 ± 20.9	< 0.001*

*VEGF* Vascular endothelial growth factor

^*^P < 0.05

**Table 3:** Plasma apelin and VEGF levels at 4–6 weeks with mild and severe ROP infants

Levels	Mild ROP group (*n* = 11)	Severe ROP group (*n* = 22)	*P*-value
apelin (pg/mL)	75.4 ± 17.7	92.4 ± 20.7	0.027*
VEGF (pg/mL)	129.0 ± 25.4	109.7 ± 23.7	0.039*

*VEGF* Vascular endothelial growth factor

^*^p < 0.05

### Prediction of ROP

**Fig. 6:**
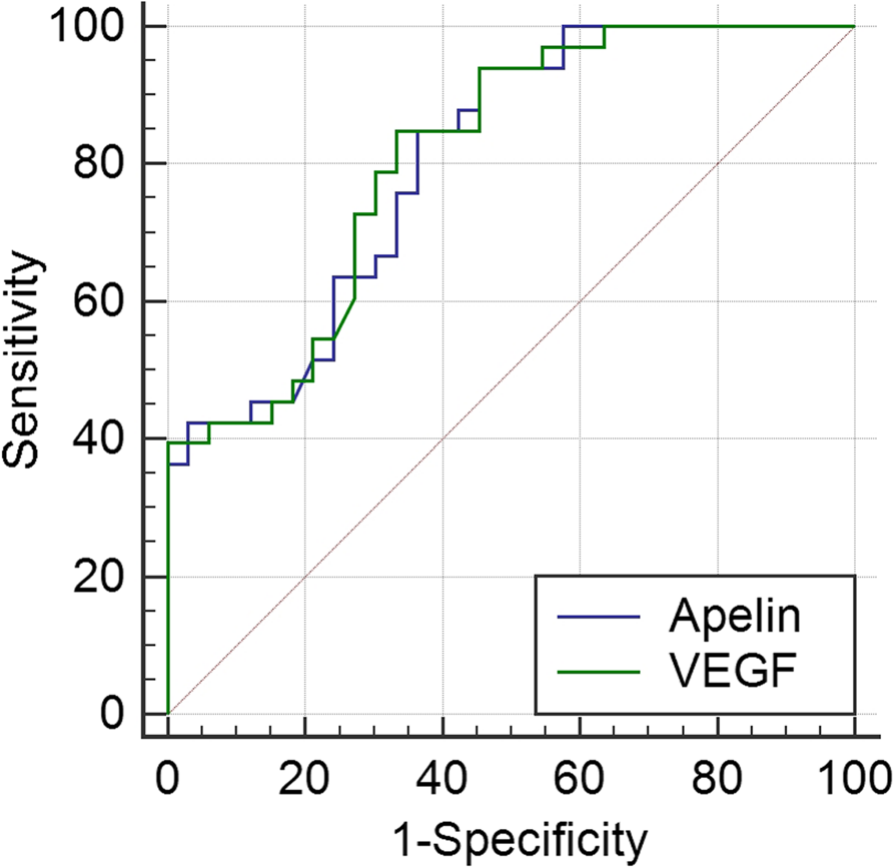
Receiver operating characteristic curve of apelin-13 and VEGF for prediction of ROP in preterm infants. The Y-axis represents sensitivity, and the X-axis represents the false-positive rate. ROP, retinopathy of prematurity; VEGF, vascular endothelial growth factor

The ROC curve was plotted to assess the appropriate values of apelin-13 and VEGF for the diagnosis of ROP (Table 4; Fig. 6). The ROC curve for apelin-13 as the indicator of ROP showed an area under the curve (AUC) of 0.804 (95% confidence interval [Cl], 0.702–0.907; *P* < 0.001), and a cut-off value of 119.6 pg/mL yielded a sensitivity of 84.8% and a specificity of 63.6%. The ROC curve for VEGF as the indicator of ROP showed an AUC of 0.810 (95% Cl, 0.695–0.896; *P* < 0.001), and a cut-off value of 84.3 pg/mL yielded a sensitivity of 84.8% and a specificity of 66.7%.

**Table 4:** ROC curve analysis results of apelin and VEGF level diagnosis of ROP

Indexes	AUC	95% CI	P	Criterion	Sensitivity	Specificity	YI
Apelin	0.804	0.702–0.907	0.000*	119.6	0.848	0.636	0.485
VEGF	0.810	0.695–0.896	0.000*	84.3	0.848	0.667	0.515

*AUC* Area under the curve, *YI* Youden’s indx

^*^P < 0.05

## Discussion

This study demonstrated that systemic apelin-13 and VEGF may play a role in assisting the diagnosis of ROP with a relatively high sensitivity and specificity and that systemic apelin-13 concentrations are negatively correlated with systemic VEGF concentrations in infants with ROP.

Improvements in our understanding of the molecular basis of diseases and advances in technology have fuelled the search for novel biomarkers for many diseases. Biomarkers can potentially improve our ability to identify, manage, or prevent a wide range of conditions that jeopardise patient health. Biomarkers are mostly used to identify the presence of a disease, selectively treat the population at greatest risk, and provide a low risk–benefit ratio [14].

Preterm infants usually require supplemental oxygen during post-natal treatment. Exposure to relatively high oxygen levels delays the formation of retinal blood vessels, causing vascular occlusion. This stage usually occurs at 30–32 weeks of gestation. When blood vessels resume growth, new abnormal blood vessels are formed by the involvement of multiple cytokines [15]. Animal models of oxygen-induced retinopathy have shown that VEGF is a key player in the pathogenesis of ROP [16]. An in vivo study on ROP has found a retinal VEGF pattern consistent with that detected in in vitro studies [17]. Concurrently, animal studies on retinal endothelial cell lines have demonstrated that apelin-13 has angiogenic activity [18, 19]. Many animal studies and a small number of human studies have demonstrated that apelin-13 is associated with the occurrence of ROP [7–9]. Thus, systemic apelin-13 and VEGF may be meaningful biomarkers to aid in the diagnosis of ROP [9, 20].

Currently, there is a consensus on the inverse correlation between the incidence of ROP and gestational age and birth weight. Given the different manifestations of ROP and various external factors, neonatal birth conditions and peri-natal treatment strategies, such as oxygen supply and blood transfusion strategies, must be given appropriate consideration in the neonatal intensive care unit [21, 22]. In our study, the selected control infants were matched in terms of gestational age and birth weight, and after comparison, sex, number of multiple births, Apgar score, oxygen supplementation, and blood transfusion were similar between the ROP and non-ROP groups. This enabled us to avoid the bias that might have been caused by the above factors in the comparison of apelin-13 and VEGF cytokines.

Our study found that preterm infants with a gestational age of < 32 weeks and birth weight of < 1500 g had higher plasma VEGF levels and lower plasma apelin-13 levels in infants with ROP than those in infants without ROP at 4–6 weeks after birth; and infants with severe ROP had higher plasma VEGF levels and lower plasma apelin-13 levels than infants with wild ROP. The results of several other in vivo studies on venous blood VEGF levels [4, 20] are consistent with the results of this study, particularly in terms of gestational age, birth weight, and sample evaluation time. However, our results are different from those reported by Pieh et al. [23] and Kandasamy et al. [24]; this may due to the different gestational ages, birth weights, and sample evaluation times of preterm infants in these studies. Differences in gestational age at birth, birth weight, and oxygen supplementation time after birth may affect cytokine levels. In terms of research on blood apelin-13 levels, both Ali et al. [9] and Cekmez et al. [2] observed significantly lower blood apelin-13 levels in the ROP group than in the control group, which is consistent with the results of our study. These two studies were also single-center studies with small-sample sizes; therefore, further research is needed. Feng et al. [3], also from our institution, reported results that are opposite to ours. They found that the VEGF levels in the ROP group were lower than those in the control group, while the apelin-13 levels were higher in the ROP group than in the control group. The reason may be that their study population included all pre-mature babies as they did not specify that the gestational age should be < 32 weeks. Another reason for the difference in the results may be due to their specimen sampling time, which was > 6 weeks after birth until approximately 3 months, as it is generally known that cytokine levels will change with time after birth [25]. The time point used for ROP screening in our study—4–6 weeks post-partum—maybe more meaningful for detecting differences in cytokine levels and, therefore, for predicting the disease. Data on the systemic levels of VEGF and apelin-13 related to ROP have been inconclusive; thus, our results provide evidence that plasma apelin-13 and VEGF levels measured at 4–6 weeks of age may play a role in the diagnosis of ROP.

In terms of the relationship between apelin-13 and VEGF, Huang et al. [26] demonstrated that apelin markedly upregulated VEGF protein expression at all selected time points after middle cerebral artery occlusion, whereas Uribesalgo et al. [27] showed that apelin, at least in part, suppressed the VEGF transcriptome in tumour vessels. In addition, Hou et al. [10] performed experiments in mice to confirm that apelin could effectively promote angiogenesis under hypoxic–ischaemic conditions, which is related to the up-regulation of VEGF. Likewise, the relationship between systemic apelin and VEGF levels in neonates with ROP is inconclusive. Zhang et al. [28] reported that there was no correlation between apelin and VEGF expression in neonates with ROP, whereas Feng et al. [3] found that the plasma levels of apelin and VEGF were negatively correlated in children with ROP. In this study, we found a negative correlation between plasma apelin-13 and VEGF levels, not only in ROP group but also in non-ROP group, which suggesting that apelin and VEGF may mutually regulate each other [29], but the mechanism is still unclear. Since the negative correlation may be related to ROP or preterm birth, further studies with large samples are needed to confirm this correlation.

In recent years, there has been an increasing interest in determining the related pathogenic mechanisms of ROP [16] and related biomarkers such as apelin-13 and VEGF, but there is insufficient conclusive evidence to support their usefulness; our research results provide some evidence. The ROC curve was used to define the best apelin-13 cut-off value for our patients, which was 119.6 pg/mL, with a sensitivity of 84.8%, specificity of 63.6%, and diagnostic accuracy of 80.4%. Another ROC curve was used to define the best VEGF cut-off value for our patients, which was 84.3 pg/mL, with a sensitivity of 84.8%, specificity of 66.7%, and diagnostic accuracy of 81.0%. This finding is in agreement with that reported by Ali et al. [11] and suggests a role of plasma apelin-13 and VEGF in the diagnosis of ROP.

This study has some limitations, including its single-center study design and relatively small number of included cases. Future multi-center studies with large samples are needed to confirm the clinical relationship between various biomarkers, such as apelin-13 and VEGF, in patients with ROP to provide innovative ideas for the screening of ROP.

## Conclusions

Differences in plasma apelin-13 and VEGF levels measured at 4–6 weeks of age may play a role in the diagnosis of ROP. This finding may provide a basis for new methods of ROP screening in the future.

## Data Availability

All data generated or analysed during this study are included in this article. Further enquiries can be directed to the corresponding author.

## Abbreviations

AUC: Area under the curve
CI: Confidence interval
ROC: Receiver operating characteristic
ROP: Retinopathy of prematurity
VEGF: Vascular endothelial growth factor
